# Introduction to special section on: current topics in cancer survivorship and work

**DOI:** 10.1007/s11764-020-00868-w

**Published:** 2020-03-23

**Authors:** A. G. E. M. de Boer, M. A. Greidanus, C. S. Dewa, S. F. A. Duijts, S. J. Tamminga

**Affiliations:** 1 Coronel Institute of Occupational Health, Amsterdam Public Health Research Institute, University of Amsterdam, Amsterdam UMC, Amsterdam, The Netherlands; 2 Departments of Psychiatry and Behavioral Sciences and Public Health Sciences, University of California, Davis, CA USA; 3 Department of Public and Occupational Health, Amsterdam Public Health Research Institute, Vrije Universiteit Amsterdam, Amsterdam UMC, Amsterdam, The Netherlands; 4 Department of Research and Development, Netherlands Comprehensive Cancer Organisation (IKNL), Utrecht, The Netherlands

**Keywords:** Cancer, Work participation, Employment, Employer, Tailored intervention, Physical limitations, Cognitive limitations

## Abstract

**Abstract:**

Work is a key contributor to quality of life and an important aspect of cancer survivorship. We call attention to current topics in cancer survivorship and work with 12 articles on cancer survivorship and work in this special section. The focus is on less studied diagnostic groups such as gastrointestinal cancer and prostate cancer, and on long-term effects of cancer diagnosis and treatment on work. Furthermore, studies are included on topics not generally studied including cognitive limitations and pain, the role of the employer on work outcomes among different types of cancer survivors and some countries not typically covered in the existing literature on work and cancer survivorship. We conclude that to improve sustainable work participation in cancer survivors, personalised, tailored interventions should be provided. A prerequisite for this is the identification of groups and individuals at high risk for adverse work outcomes. In order to develop such interventions, research involving new approaches such as matching data registries, participatory approaches and the involvement of many stakeholders and survivors with these different types of cancer diagnoses is necessary.

**Implications for Cancer Survivors:**

The goal of sustainable work participation in cancer survivors can be improved by the delivery of a personalised or risk-based tailored intervention. Furthermore, successful work outcomes often involve many stakeholders who should all be included Implications for Cancer Survivors. The goal of sustainable work participation in cancer survivors can be improved by the delivery of a personalised or risk-based tailored intervention. Furthermore, successful work outcomes often involve many stakeholders who should all be included.

## Introduction

The number of cancer survivors is growing steadily due to continuous improvements in screening and in multimodal treatment of cancer [1, 2]. Paid work is a key contributor to quality of life in cancer survivorship and important for cancer survivors, their families and society at large [3, 4]. It is associated with a higher quality of life, self-esteem, social status and is often experienced as a sign of recovery after a long period of treatment [4]. In ageing Western societies, it is also an economic necessity to stimulate work participation whenever possible because of a decreasing labour force [5].

In his 2007 inaugural article of this journal, “Optimizing cancer survivorship”, Prof. M. Feuerstein noted that work, as one of many functional outcomes, is an important topic consistent with the journal’s mission of disseminating information to improve several outcomes related to cancer survivorship [6]. Almost a decade and a half later, we have seen a rapid increase in articles and reviews on cancer survivorship and work. These articles have shown that cancer survivors do indeed have a higher risk of unemployment or no return to work compared with healthy individuals [3, 7]. Most of these studies have been limited to survivors of breast cancer, short-term work outcomes (up to 5 years) and North American or European populations [3, 7]. The most frequently studied long-term effects of cancer treatment affecting sustainable work participation include fatigue, physical problems, anxiety and depression [8]. Finally, most research have studied the effect of cancer survivorship on work from the patient’s perspective, while the viewpoint of other stakeholders, such as employers, is less well understood [9].

In this special section of the JCSU, we call attention to current topics in cancer survivorship and work. The focus of the 12 articles in this special section is on less studied diagnostic groups such as gastrointestinal cancer and prostate cancer, and on long-term effects of cancer diagnosis and treatment on work. We include research on topics not generally studied including cognitive limitations and pain, the role of the employer on work outcomes among different types of cancer survivors and some countries not typically covered in the existing literature on work and cancer survivorship.

## Return to work in less studied cancer diagnosis groups

Two articles in this special section focus on prostate [10] and gastrointestinal cancer survivors [11] which are cancer types not often included in studies on work. In a systematic review of work after prostate cancer, 12 studies reported an average return to work (RTW) of 80% and a mean sick leave duration of 32 days [10]. The authors conclude that these findings indicate a more positive RTW outcome as compared with other diagnoses. However, this might not apply to those with physically demanding or low paid jobs, comorbid conditions or poor physical functioning. Zaman et al. [11] reported on the evaluation of a tailored psychosocial work-related support intervention targeting gastrointestinal cancer survivors. The intervention is delivered by either an oncology nurse or an occupational physician with expertise in oncology, depending on the severity of symptoms at diagnosis and in the first year after diagnosis. This strategy of tailored work-related support was valued by both cancer survivors and healthcare professionals, and was found to be feasible in clinical practice. While this type of work-related support may serve as a model for other diagnoses, the timing of this intervention, prior to the onset of cancer treatment, may need to be adjusted because this timing was perceived as too early for some cancer survivors.

In a large population-based study among Japanese cancer survivors [12], it was found that 88% of cancer survivors returned to work. However, females and those with haematological cancers had a fourfold greater chance of not returning to work while temporary workers were 2.5 times more likely to not return to work. The authors conclude that those at higher risk for not returning should engage in efforts that balance cancer treatment and work, and followed more carefully post-cancer diagnosis [12]. Australian authors summarise the existing qualitative reviews and recent studies, and examine cancer survivors’ motivations for and experiences of RTW [13]. Important qualitative studies investigated experiences of cancer survivors concerning work. Some important perceptions were identified, including the “meaning of work”, “intrinsic value of work”, “disclosure of cancer at work”, “coping with limitations”, “responses from employers and co-workers”, “overall work culture”, “financial issues” and “social support”. Given these experiences, future, effective RTW interventions should directly address these specific psychosocial and work-related issues [13].

### Long-term work effects of cancer treatment on paid work

Three articles in this special section offer insights into cancer’s long-term impact on paid work. De Boer and colleagues from the CANWON group that study work in cancer survivors with many different types of cancer [14], report on a systematic literature review. The review identified 21 studies investigating cancer survivors’ work outcomes 2 years or more post-diagnosis [15]. They report that approximately 73% of the survivors were at work 2–14 years post-diagnosis. Their meta-analysis indicated that a lower probability of working over the long-run was significantly associated with greater age, lower income, receipt of chemotherapy, presence of comorbidities and a lack of work accommodations at the workplace [15].

Two recent articles not included in this special section but published in this journal also contribute to this body of literature. Using a Dutch sample of 1974 breast cancer survivors, Tamminga et al. [16] reported that 36% of the cancer survivors experienced adverse work outcomes 5–10 years post-diagnosis. Adverse work outcome was defined as either underemployment or labour market exit [16]. Their findings corroborate Boer and colleagues [15], while adding that perceived value of work, higher work ability, familial responsibilities and feeling supported at work, also significantly positively affected long-term work outcomes.

In a study using Israeli census and cancer registry data, variability by cancer type was observed in the unemployment rates of cancer survivors 10 years following treatment. This outcome ranged from 19% for testicular cancer to 44% for central nervous system malignancies [17]. This study highlights the need for more research on longer term employment by cancer type and long-term and late treatment side effects.

## Cognitive functioning and pain

Two articles on cognitive functioning and work in cancer survivors were also included in this special section. Ehrenstein et al. [18] explored the longitudinal association between type of cancer treatment and cognitive symptoms in cancer survivors once back at work. While data on 330 working heterogeneous cancer survivors showed that patients who received chemotherapy reported comparable memory function as those receiving locoregional treatment, executive function differed. Executive functions were significantly lower for cancer survivors who received chemotherapy. The authors conclude that cancer survivors who are at work may require some type of cognitive function management irrespective of cancer treatment exposure. However, a more careful consideration of executive function in those with exposure to chemotherapy is also warranted.

As with Ehrenstrein et al., Klaver et al. [19] also focused on cancer-related cognitive problems in working cancer of non-CNS cancer survivors. Results of qualitative data from three focus groups indicated that 23 cancer survivors (mainly with a diagnosis of breast or cervix cancer) actually used several different strategies (e.g., applying practical adjustments, re-organisation of work and accepting limitations) to cope with cancer-related cognitive problems. Two focus groups with professionals indicated that they use similar approaches in their attempt at supporting cancer survivors facing these problems. The authors state that support for working cancer survivors who experience cancer-related cognitive problems is important, as it might increase work participation over the long-term.

In a cross-sectional study focusing on pain, Cox-Martin et al. [20] reported on 1702 heterogeneous cancer survivors post-treatment of which 32% were breast cancer survivors. The authors indicated that nearly 17% of the working-age cancer survivors reported cancer-related pain. Among those who experienced pain, the majority were female, white, non-Hispanic, married/partnered and non-employed, with breast as the most common cancer site. As expected, cancer-related pain decreased the odds of being employed, however only in female survivors [20]. The authors suggest that that women, in particular, may benefit from interventions that target persistent cancer-related pain.

## The role of the employer

The remaining two articles in this special section address the employer’s perspective on the RTW in cancer survivors. This is an understudied perspective when considering optimization of work participation in those affected by cancer. The article by De Rijk et al. [21] from the CANWON group [14] presents the findings of interviews from nine countries. While mostly European countries, the study aims to obtain a better understanding of the employers’ experiences using “good practices” related to RTW in employees diagnosed and treated for heterogeneous types of cancer [21]. The authors conclude that assisting a cancer survivor RTW is a dynamic process, consisting of different phases. Interestingly, both “good practice” and the employers’ needs showed strong similarities across the different countries.

The article by Greidanus et al. [22] describes, to the best of our knowledge, the first intervention that solely targets employers in relation to cancer survivorship, i.e. the MiLES intervention. This web-based intervention was developed in close collaboration with several employers and other stakeholders, in order to meet employers’ needs and increase it feasibly in actual practice [22]. Although its effectiveness on various work outcomes in several types of cancer survivors remains to be empirically determined, the MiLES intervention does provide the description of a potentially useful approach based on the employer’s perspective.

## Future research and practice

The articles included in this special section and the recent literature on cancer and work in general, it is likely that the goal of sustainable work participation in cancer survivors can be improved by identifying groups of various cancer survivors who are at high risk for adverse work outcomes. This special section also highlights the importance of understanding individual and work-related psychosocial factors that contribute to the risk of unemployment among cancer survivors. These data are essential for the delivery of a personalised or risk-based tailored intervention.

For example, a cancer survivor at high risk of adverse work outcomes due to cognitive problems might receive a personalised intervention including cognitive training delivered by a (neuro-) psychologist or professional in a healthcare setting, as suggested by Ehrenstein et al. [18] and Klaver et al. [19]. While another cancer survivor at high risk because of a lack of employer support might benefit from an intervention that would involve the employer using a web-based intervention or communication skills training approach, as suggested by Greidanus et al. [22] and de Rijk et al. [21]. Lastly, a third cancer survivor who needs to work to support her family, experiencing pain and number of health complications, might benefit from a multidisciplinary team as proposed by Zaman et al. [11]. In each case given what findings strongly imply, it is also important to communicate with the workplace.

Many different factors have been associated with whether or not a cancer survivor, if so desired, is able to be involved in paid work. Some of these factors may not be immediately amendable to change such a policy related to work in cancer survivors. However, many long-term and late effects experienced by cancer survivors can be modified. Providers, cancer survivors and employers need to be made more aware of this possibility. Successful work outcomes often involve many stakeholders with at times opposing views and interests [23]. These also need to be recognised and when possible made explicit and be resolved.

Research directed at cancer survivors with low socio-economic status, those involved in diverse work situations and those from ethnic minorities need to be expanded. We should also pay greater attention to the effects of cognitive factors and pain on the ability to complete required work tasks. The use of different methodologies in research in this area may be helpful as well, for example matching registry data with a well-defined target group of cancer survivors (e.g. Kvillemo et al. [24] and de Boer [14]). Designing interventions with greater input from cancer survivors as well as the many stakeholders involved in the process of actually returning and retaining cancer survivors in work should prove helpful [25]. Specifically, the role of stakeholders, such as family members, healthcare professionals and employers, all need further study in order to determine their influence on work outcomes [26]. Finally, future research should focus on cancer types that are still understudied in cancer and work research, such as haematological, gastrointestinal cancers and other rare cancer types, as suggested by Butow et al. [13]. This research would provide evidence-based interventions for various cancer types and work outcomes. This information should provide a set of more specific interventions to improve work participation.

## Conclusion

In this special section, we have focussed on current topics in cancer survivorship and work. We conclude that, to improve sustainable work participation in cancer survivors, personalised, tailored interventions should be provided. A prerequisite for this is the identification of groups and individuals at high risk for adverse work outcomes. In order to develop such interventions, research involving new approaches such as matching data registries, participatory approaches and the involvement of many stakeholders and survivors with these different types of cancer diagnoses is necessary.
